# The Utility of Short-Interval Repeat Computed Tomography Angiogram After Blunt Cerebrovascular Injury in Adults

**DOI:** 10.7759/cureus.9968

**Published:** 2020-08-23

**Authors:** Zaid Aljuboori, Kimberly Meyer, Dale Ding

**Affiliations:** 1 Neurological Surgery, University of Louisville School of Medicine, Louisville, USA; 2 Neurological Surgery, University of Louisville, Louisville, USA

**Keywords:** trauma, dissection, vascular, vertebral, carotid

## Abstract

Introduction

Blunt cerebrovascular injury (BCVI) can lead to thromboembolic events. The necessity of short-interval noninvasive vascular imaging after the initial diagnosis is controversial. This retrospective cohort assesses the utility of short-interval computed tomography angiography (CTA) after an initial diagnosis of BCVI.

Methods

We retrospectively reviewed patients with BCVI managed at our institution from 2016 to 2019 who underwent a short-interval (one to three weeks) repeat CTA after initial diagnosis. We excluded patients with age <18 years, penetrating injury, and previous neck irradiation. We collected baseline data and performed logistic regression to identify predictors of BCVI imaging outcomes.

Results

The study cohort comprised 38 patients with a mean age of 45 years with 68% males. Motor vehicle crash (MVC) was the most common mechanism of 79% injury. Unilateral vertebral artery (VA) was the most common vessel that is 66% injured, and grade I 36% was the most common dissection on initial CTA. Grades III and IV dissection 33% were the most common in the short-interval CTA. Shift analysis showed a significant improvement in Biffl grades I on the short-interval CTA (p = 0.0001). Antiplatelet therapy or anticoagulation (AC) was administered to 82% of patients after the initial diagnosis. The rates of early (<2 weeks) and delayed (two weeks to three months) ischemia were 5% and 0%, respectively, and endovascular stenting was performed in 8%.

Conclusion

BCVI grades I and II are more frequent than high-grade injuries. Short-interval non-invasive vascular imaging can detect changes of BCVI which can affect the management paradigm. It also can select patients who will benefit from endovascular intervention and avoid stroke. Besides, the short-interval non-invasive vascular imaging will not incur additional cost or increase exposure to radiation.

## Introduction

Blunt cerebrovascular injury (BCVI) is an injury of the carotid and/or vertebral arteries (VAs) due to non-penetrating trauma. It is usually associated with fractures of the skull base, cervical spine, or first rib. Biffl et al. described the most commonly used classification scheme for BVCI [1]. The trauma can cause an intimal tear and, in some cases, leads to pseudoaneurysm formation [2-5]. Intimal injury can lead to thrombosis and vessel occlusion or release of emboli, and both can lead to devastating stroke [6-8]. Antiplatelet or anticoagulation (AC) is usually used to prevent such complications, and in some cases, endovascular intervention is needed [9]. 

There is controversy concerning the need for short-interval follow-up vascular imaging (e.g., computed tomography angiography, CTA) after the initial diagnosis. Some authors suggested that it can clarify the diagnosis where the initial CTA was confounded by vessel spasm [6]. Others suggested that it may not be beneficial in high-grade BCVI, as most injuries do not resolve. Also, it may incur an unnecessary additional cost and radiation [10]. We report our analysis on the utility of short-interval CTA after an initial diagnosis of BCVI.

## Materials and methods

We retrospectively reviewed consecutive patients with BCVI managed at the University of Louisville from 2016 to 2019 who underwent short-interval (one to three weeks) CTA after initial diagnosis. The exclusion criteria were age <18 years, penetrating injury, a diagnosis of BCVI with no short-interval CTA, and previous neck irradiation. We collected the patients’ demographics, mechanism of injury, presence of cervical spine or first rib fractures, grade of dissection on initial and repeat CTA, use of antiplatelet or AC, the incidence of stroke, and need for vascular intervention.

Statistical analysis

We used the mean with standard deviation to summarize continuous variables; counts and percentages summarized categorical variables. We used Chi-square to compare categorical outcomes and the ordered logistic regression for ordinal outcomes. Multinomial logistic regression was used to identify predictors of imaging outcomes of vascular injury. All tests were two-sided with a significance level of 0.05. Statistical data analysis was performed in Stata 13 (StataCorp, College Station, USA).

## Results

**Figure 1:**
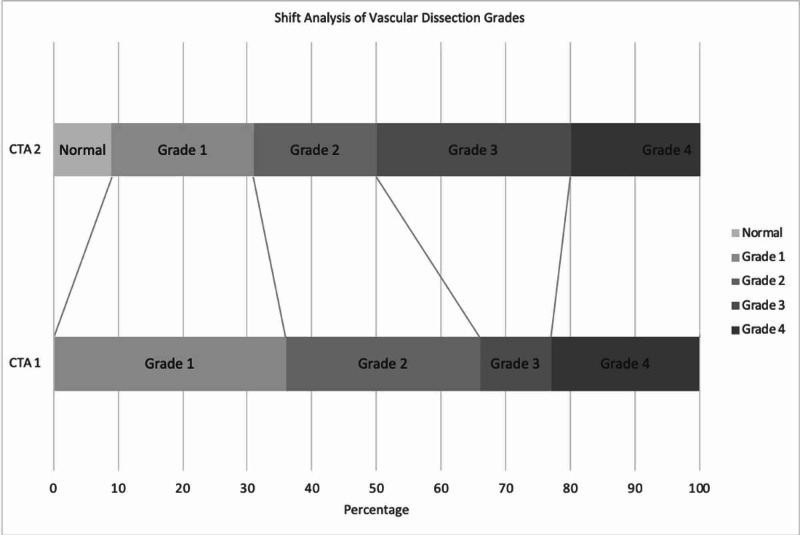
A bar graph that shows the shift analysis of vascular dissection grade on repeat (second CTA) imaging CTA: computed tomography angiogram.

The total number of patients with a diagnosis of BCVI was 205, but only 38 (15%) had short-interval CTA (because of differences in the management strategy of BCVI among neurosurgeons at our institution). For patients (N = 38) who had a short-interval CTA, the mean age was 45 years for males (68%). MVC was the most common mechanism of injury (79%), and 89% of patients had cervical spine fractures (Table 1). Unilateral VA was the most common vessel (66%) injured and grade I (36%) was the most common dissection on initial CTA. Grades III and IV dissection (33%) were the most common on the short-interval repeat CTA (Table 2). Shift analysis showed a significant improvement in Biffl grades I on the short-interval repeat CTA (p = 0.0001, Figure 1). In addition, Biffl grade I injuries were more likely to improve (relative risk ratio [RRR] = 3.6, CI 95% (1.02-13.1), p = 0.04), whereas grade IV injuries were more likely to be stable (RRR = 33, CI 95% (2.9-374), p = 0.005). Antiplatelet therapy or AC was administered to 82% of patients after the initial diagnosis. Ten BCVIs (26%) were resolved on repeat imaging (Table 3). The rates of early (<2 weeks) and delayed (2 weeks to 3 months) ischemia were 5% and 0%, respectively, and endovascular stenting was performed in 8% of patients. 

**Table 1:** Patients demographics AC: anticoagulation, ASA: aspirin, CTA: computed tomography angiogram, MVC: motor vehicle crash, FFH: fall from height.

Variable		Percentage
Gender	Males	68%
Mechanism of injury	Fall	21%
	MVC	79%
Cervical spine fractures		89%
First rib fractures		16%
Early stroke		5%
Delayed stroke		0%
Endovascular intervention		8%
Received ASA or AC between first and second CTA		82%
Received ASA or AC after second CTA		82%

 

**Table 2:** Percentage of vascular injuries VA: vertebral artery, ICA: internal carotid artery.

Variable	Number of dissections	Percentage
VA (unilateral)	25	66%
VA (bilateral)	4	11%
Total VA	33	70%
ICA (unilateral)	6	16%
ICA (bilateral)	4	11%
Total ICA	14	30%

 

 

 

**Table 3:** Imaging outcomes of vascular injuries on second and third CTA CTA: computed tomography angiogram. Normalized = normal findings on CTA. Improved = increased flow, increased diameter, smaller pseudoaneurysm, etc. Stable = similar characteristics to previous imaging. Worsened = decreased flow, decreased diameter, larger pseudoaneurysm, etc.

Number of patients	Second CTA	Third CTA
Total	38	16
Normalized	9 (24%)	1 (6%)
Improved	2 (5%)	6 (38%)
Stabilized	14 (37%)	9 (56%)
Worsened	13 (34%)	0%

## Discussion

BCVI are managed medically with antiplatelet or anticoagulants, with endovascular stenting reserved for symptomatic or worsening stenosis, and the severity of the injury correlates with the risk of stroke [8,9]. In this study, the short-interval (one to three weeks) repeat CTA revealed that (65%) of grade I dissection had resolved, and a sizable percentage of grade II (57%) dissection had progressed to grades III and IV. These findings echo what Wagenaar et al. reported as they showed that grades I and II had a higher chance for improvement (56% and 18%), respectively, compared to grades III and IV (8% and 2%), respectively [10]. Three patients (8%) with ICA dissection needed an early (within two weeks of injury) endovascular stenting because of a worsened dissection in one case and ischemic complications in the other two. Notably, all three patients were on ASA. In retrospect, the two patients who had ischemic complications could have benefited from an earlier (< 10 days) repeat CTA as it might have led to earlier detection of the worsening of the ICA dissection and prevented the ischemic complications. The delayed CTA (8-16 weeks) showed only 6% of patients had a resolution of their dissections with the remaining having either improved to a lower grade (38%) or remained stable (56%). Notably, none of the patients required a delayed endovascular intervention, and there were no delayed strokes (two weeks to three months) within the cohort. Eighty-two percent of patients were treated with ASA or oral AC, while the remaining (18%) did not receive during the early phase of injury because of planned surgical intervention during hospitalization.

The short-interval CTA is beneficial to detect changes in the grade of the dissection as we showed that most BCVI resolved or worsen in the early phase of the injury. It will also enable the treating physician to manage any worsening hastily, especially for patients who need endovascular intervention, which can prevent the devastation caused by a stroke. For patients with resolved dissection, AC or ASA can be stopped. From a cost-saving perspective, the resolution of the dissection on the short-interval CTA will obviate the need for a delayed CTA, which will not incur additional cost or increase exposure to radiation for this subgroup of patients. Patients with persistent dissection on the short-interval CTA will need further observation with vascular imaging. Although this will add cost and radiation exposure, the detection and treatment of any injury progression on the short-interval CTA can prevent the disability and financial consequences of a stroke. Therefore, we recommend a short-interval CTA within one to three weeks of the initial diagnosis or even earlier in patients with ICA dissection.

Limitations

One should keep in mind that our study is retrospective with relatively small sample size and the potential for selection bias. Also, interobserver variability can exist as different radiologists read different imaging studies. Therefore, additional studies are necessary to clarify the role of short-interval non-invasive vascular imaging in the management of BCVI.

## Conclusions

Low-grade BCVI is more frequent than high-grade injuries. Short-interval non-invasive vascular imaging can identify changes in BCVI which can alter the management plan. It also can identify patients who will benefit from endovascular intervention and prevent stroke. Besides, the short-interval non-invasive vascular imaging will not incur additional cost or increase exposure to radiation. 
